# Effect of Paliperidone Combined with Sertraline in the Treatment of Schizophrenia and its Influence on Serum Neurofunctional Related Factors

**DOI:** 10.31083/AP38775

**Published:** 2025-02-28

**Authors:** Meng Gu, Zhilian Pi, Long Zhu, Jun Zhang

**Affiliations:** ^1^Department of Psychiatry, Jiande Fourth People’s Hospital, 311612 Jiande, Zhejiang, China; ^2^Department of Gerontology, Huangshi Psychiatric Hospital, 435000 Huangshi, Hubei, China

**Keywords:** schizophrenia, paliperidone, sertraline, neuroregulatory protein

## Abstract

**Objective:**

This study aimed to evaluate the efficacy of paliperidone combined with sertraline in treating schizophrenia (SCZ) and its effect on serum neurofunctional factors.

**Methods:**

A retrospective analysis was conducted on SCZ patients admitted between June 2020 and June 2021. Initially, 80 patients were treated with paliperidone, while 36 received a combination of paliperidone and sertraline. Propensity score matching based on 3 covariates resulted in 2 groups: the control group (paliperidone alone, n = 36) and the observation group (paliperidone + sertraline, n = 36). The clinical efficacy, adverse reactions, quality of life scores, serum biomarkers levels related to nerve and liver function, and anxiety and depression levels were compared between the 2 groups.

**Results:**

The observation group demonstrated higher total effectiveness than the control group (*p* = 0.011). Post-treatment, the scores of all dimensions of quality of life in both groups were improved, and the observation group was higher than the control group (*p* < 0.001). Post-treatment, the observation group exhibited lower neuron-specific enolase (NSE) and higher neuregulin 1 (NRG1) levels than the control group (*p* < 0.001). The levels of aminotransferase (AST), total bilirubin (TBiL) and alanine aminotransferase (ALT) increased in both groups post-treatment (*p* < 0.001). The levels of tumor necrosis factor-α (TNF-α), interleukin-6 (IL-6), and interleukin-2 (IL-2) decreased in both groups post-treatment, and the observation group had lower levels of these cytokines compared to the control group after treatment (*p* < 0.001). Post-treatment, Hamilton Anxiety Scale (HAMA) score and Hamilton Depression Scale (HAMD) score decreased in both groups, with the observation group showing lower scores than the control group (*p* < 0.001). The changes in the scores of various dimensions of quality of life, HAMA and HAMD scores, neurofunctional factors and inflammatory markers levels in the observation group were greater than those in the control group (*p* < 0.001). There were no serious side effects during and after treatment in both groups.

**Conclusions:**

Paliperidone combined with sertraline effectively improves serum neuregulin levels in SCZ patients, alleviates negative emotional effects without causing liver or kidney damage, and demonstrates excellent clinical efficacy and safety.

## Main Points

1. Paliperidone combined with sertraline can effectively alleviate serum neuregulin levels in patients with schizophrenia.

2. Paliperidone combined with sertraline improves the negative emotional effects on patients and does not cause liver or kidney function damage.

3. Compared with traditional drug treatment, the clinical efficacy and safety of paliperidone combined with sertraline is promising.

## 1. Introduction

Schizophrenia (SCZ) is a destructive neuropsychiatric disorder, belonging to the group of chronic sensory, behavioral, and emotional disorders, that is usually accompanied by mental decline, mental disability, and other diseases, and is prone to relapse [1, 2, 3, 4]. The main clinical manifestations are abnormalities in thinking, sensory perception, emotional experience and will behavior, as well as the incoordination of emotional responses and mental activities, which often damage the social function of patients, placing a heavy disease burden on the family and society. Timely treatment can effectively control the development of the disease, preventing patients from losing basic social and life skills, and improving patients’ long-term quality of life [5].

Since changes in neurotransmitter levels are one of the key findings of SCZ pathology, antipsychotic drug treatment is considered one of the most important building blocks of SCZ management [6, 7, 8]. Positive symptoms and cognitive deficits in patients with SCZ can be alleviated through drug therapy, thereby reducing the frequency of symptoms and controlling the progression of the disease [9]. Among these, paliperidone is an atypical antipsychotic drug commonly used in the clinical treatment of SCZ, which can adjust the sleep structure and circadian rhythm of patients, regulate adverse emotions, maintain normal function of the nervous system, and promote recovery of cognitive and emotional functions, effectively controlling disease symptoms [10].

Sertraline is a commonly used antidepressant drug in clinical practice, belonging to the class of selective serotonin reuptake inhibitors, which have a weak affinity for dopaminergic and adrenergic receptors and can directly adjust neurotransmitters, reduce the level of inflammatory factors, and control and alleviate the condition to a certain extent [11]. Sertraline can effectively improve hallucinations, anxiety, sleep disorder hyposmia, and other related symptoms, with fewer clinical adverse reactions, but its single use has little impact on patients’ cognitive function and related neurological function indicators [11]. However, there have been few studies on paliperidone combined with sertraline in the treatment of SCZ. Many scholars at home and abroad have conducted in-depth studies on the relationship between schizophrenia and cytokines, especially regarding the serum interleukin level of SCZ patients, but the conclusions are inconsistent.

This study aims to analyze the observed effects of paliperidone combined with sertraline in the treatment of SCZ patients and its influence on serum factors related to nerve function, in order to provide valuable clinical insights into treatment strategies for patients with SCZ, and better improve clinical outcomes.

## 2. Materials and Methods

### 2.1 General Information

Patients with SCZ admitted to our hospital from June, 2020 to June, 2021 were retrospectively collected as research subjects. Of these, 80 patients were treated with paliperidone and 36 patients were treated with paliperidone combined with sertraline. 1:1 propensity score matching (PSM) was used to balance the influence of confounding factors among the groups, and three covariables were selected for 1:1 matching (gender, age, positive and negative symptom scale (PANSS) score). Finally, 36 cases of paliperidone combined with sertraline and 36 cases of paliperidone were successfully matched. This study was reviewed and approved by the medical ethics committee of the hospital (number: 2023001-03).

Inclusion criteria: (1) Relevant examinations were completed after admission and the patients were in line with the International Classification of Diseases, 10th Revision (ICD-10) Classification of Mental and Behavioral Disorders [12]. (2) The patient had not taken paliperidone or sertraline in the recent period. (3) PANSS score was ≥60 points. (4) No congenital mental disorders were present and the patient had adequate communication skills. (5) Patient data were complete.

Exclusion criteria: (1) Patients with heart, liver, lung, and other malignant tumors. (2) Allergies to paliperidone, sertraline, or other drugs. (3) Patients with serious self-injury or severe suicidal tendencies. (4) Patients with organic diseases. (5) A family history of epilepsy. (6) Accompanying central nervous system disease. (7) Chronic alcoholism. (8) Lactating and pregnant women.

### 2.2 Therapeutic Method

Control group patients received paliperidone (SFDA approval number H20203265, specification: 3 mg, Jiangsu Haosen Pharmaceutical Group Co., Ltd., Lianyungang, China) as a daily morning dose, 3 mg/d, according to the patient’s tolerance to the drug and their own ability to adjust the dose, increasing to 6–12 mg/d, once every morning. The observation group were given sertraline (SFDA approval number H20060364, specification: 50 mg, Guangzhou Baiyunshan Guanghua Pharmaceutical Co., Ltd., Guangzhou, China) in addition to the drug regimen of the control group, once a day, 50 mg each time. One week later, according to the patient’s ability to increase the drug dose, the dose was increased to 100 mg/d. The dose could be further increased or decreased as appropriate, with the maximum dose not to exceed 200 mg. Both groups were treated for 8 weeks.

### 2.3 Clinical Efficacy

The clinical efficacy of the two groups after treatment was compared. The evaluation criteria were as follows [13]: Cured: after 12 weeks of treatment, patients’ PANSS scores were more than 75% lower than before treatment. Significant improvement: the PANSS score decreased by 50% or more and less than 75% after treatment. Improvement: the PANSS score decreased by 25% or more and less than 50% after treatment. Ineffective: the PANSS score decreased by less than 25% after treatment. Total response rate = (cure + significant improvement + improvement) cases/total cases × 100%.

### 2.4 Quality of Life

To compare the quality of life before and after treatment between the two groups, schizophrenia quality of life scale (SQLS) was used [14], which included assessment of material life, physical function, psychological function, and social function. Each dimension uses a percentage system. The higher the score, the better the quality of life of patients.

### 2.5 Detection of Factors Related to Nerve Function and Liver Function

Before and after treatment, 6 mL of fasting elbow vein blood was collected from the two groups, and the supernatant was extracted after centrifugation. The levels of neuron-specific enolase (NSE) (ml060406, Shanghai Enzyme-linked Biotechnology Co., Ltd., Shanghai, China), neuregulin 1 (NRG1) (ml024389, Shanghai Enzyme-linked Biotechnology Co., Ltd., Shanghai, China), glutamic oxalic aminotransferase (AST) (ml095196, Shanghai Enzyme-linked Biotechnology Co., Ltd., Shanghai, China), total bilirubin (TBiL) (M1224L, Shanghai Enzyme-linked Biotechnology Co., Ltd., Shanghai, China), and alanine aminotransferase (ALT) (ml095164, Shanghai Enzyme-linked Biotechnology Co., Ltd., Shanghai, China) levels were measured using enzyme-related immunosorbent assay. Liver function index AST normal value: 0~50 U/L; TBiL normal value: 5.0~21.0 µmol/L; ALT normal value: 0~50 U/L. A detection value of each index exceeding the upper limit of the normal value indicates abnormal liver function. For obvious liver function abnormalities, that is, when the value is more than 2 to 3 times the normal value, liver protection drugs should be used.

### 2.6 Levels of Inflammatory Cytokines

Five milliliters of blood were drawn in a test tube without anticoagulants, coagulated naturally at room temperature for 20 min, then centrifuged at 3000 r/min to isolate the serum, which was stored at –80 °C until it was removed for cytokine analysis. Tumor necrosis factor α (TNF-α) (ab309419, Abcam, Cambridge, UK), interleukin-6 (IL-6) (ab178013, Abcam, Cambridge, UK), and interleukin-2 (IL-2) (ab100566, Abcam, Cambridge, UK) levels were measured using enzyme-linked immunosorbent assay (ELISA).

### 2.7 Anxiety and Depression Levels

The Hamilton Anxiety Scale (HAMA) and the Hamilton Depression Scale (HAMD) were used to evaluate the anxiety and depression levels of the two groups of patients before and after the intervention. The scores of both are inversely proportional to their levels, with the higher the score, the more serious the anxiety and depression levels of the patients [15].

### 2.8 Differences in Scores and Percentage Changes between Groups

Differences in scores and percentage changes for measurements were calculated to determine the baseline changes and the difference between groups. Difference in score = score after treatment – score before treatment. Percentage change = [((measurement after treatment – measurement before treatment)/measurement Before treatment) × 100]. Comparisons were made between groups according to these differences in scores and percentage changes.

### 2.9 Adverse Reactions

By referring to the adverse reaction return sheet, the adverse reactions in the course of clinical treatment were statistically analyzed and the adverse reactions were recorded.

### 2.10 Statistical Analysis

SPSS v26.0 software (IBM, Armonk, NY, USA) was used for statistical analysis. Continuous data were represented by mean ± standard deviation (SD), as used for SQLS and HAMA scores. The independent sample *t*-test was used for comparison between groups, and the paired *t*-test was used for comparison of before and after treatment. Categorical data were represented by n (%), and the comparison between groups was tested by the χ^2^ test. *p* < 0.05 was considered to be statistically significant.

## 3. Results

### 3.1 Comparison of General Information between the Two Groups

Observation group: 19 males and 17 females, ranging in age from 26 to 40 years, with a mean age of (32.44 ± 3.22) years, the course of disease was (11.13 ± 2.12) years, and the total PANSS score was (75.63 ± 6.37). Control group: 21 males and 15 females, ranging in age from 25 to 39 years, with a mean age of (32.19 ± 3.79) years, the course of disease was (12.01 ± 2.27) years, and the total PANSS score was (75.70 ± 6.87). There was no significant difference in general data (gender, age, course of disease, and PANSS score) between the two groups (*p* = 0.635, *p* = 0.764, *p* = 0.094, *p* = 0.964).

### 3.2 Comparison of Clinical Efficacy between the Two Groups

The total effective rate of the observation group [n = 34 (94.44%)] was significantly higher than that of control group [n = 26 (72.22%)] (*p* = 0.011), as shown in Table 1.

**Table 1.** **Comparison of clinical efficacy between the two groups [n (%)]**.

Group	Cured	Significant Improvement	Improvement	Ineffective	Total Response Rate
Observation group (n = 36)	20 (55.56)	9 (25.00)	5 (13.89)	2 (5.56)	34 (94.44)
Control group (n = 36)	11 (30.56)	7 (19.44)	8 (22.22)	10 (27.78)	26 (72.22)
*p*-value					0.011

### 3.3 Comparison of SQLS Scores between the Two Groups

Before treatment, there were no significant differences in the scores for material life, physical function, psychological function, and social function between the two groups (*p* = 0.624, *p* = 0.755, *p* = 0.810, *p* = 0.617). After treatment, the scores for material life, physical function, psychological function, and social function in two groups were higher than before treatment (*p* < 0.001), and in the observation group they were higher than in the control group (*p* < 0.001), as shown in Table 2.

**Table 2.** **Comparison of SQLS scores between the two groups ($\bar{x}$ ± s)**.

Group	Material Life		Physical Function		Psychological Function		Social Function	
	Before Treatment	After Treatment	Before Treatment	After Treatment	Before Treatment	After Treatment	Before Treatment	After Treatment
Observation group (n = 36)	51.85 ± 3.48	59.56 ± 4.11^*#^	51.11 ± 3.18	59.74 ± 3.75^*#^	50.93 ± 3.73	59.81 ± 4.32^*#^	48.89 ± 3.22	58.47 ± 3.45^*#^
Control group (n = 36)	51.47 ± 3.05	55.73 ± 3.54^*^	51.34 ± 3.06	55.57 ± 3.20^*^	50.73 ± 3.30	55.24 ± 3.67^*^	48.47 ± 3.85	54.54 ± 3.05^*^
*p*-value	0.624	<0.001	0.755	<0.001	0.810	<0.001	0.617	<0.001

Compared with before treatment, ^*^p < 0.001; compared with the control group, ^#^*p* < 0.001. SQLS, Schizophrenia Quality of Life Scale.

### 3.4 Comparison of Neural Function Related Factors between the Two Groups

Before treatment, there were no significant differences in NSE and NRG1 levels between the two groups (*p* = 0.793, *p* = 0.689). After treatment, NSE was significantly decreased in both groups (*p* < 0.001), and in the observation group it was significantly lower than in the control group (*p* < 0.001). NRG1 was significantly increased in both groups (*p* < 0.001), and in the observation group it was significantly higher than in the control group (*p* < 0.001), as shown in Table 3.

**Table 3.** **Comparison of factors related to neural function between the two groups ($\bar{x}$ ± s)**.

Group	NSE (µg/L)		NRG1 (ng/L)	
	Before Treatment	After Treatment	Before Treatment	After Treatment
Observation group (n = 36)	25.36 ± 5.15	13.34 ± 2.91^*#^	10.73 ± 2.32	18.47 ± 3.27^*#^
Control group (n = 36)	25.69 ± 5.46	16.11 ± 3.39^*^	10.93 ± 1.88	14.89 ± 3.46^*^
*p*-value	0.793	<0.001	0.689	<0.001

Compared with before treatment, ^*^p < 0.001; compared with the control group, ^#^*p* < 0.001. NSE, neuron-specific enolase; NRG1, neuregulin 1.

### 3.5 Comparison of Liver Function Indexes between the Two Groups

Before treatment, there were no significant differences in liver function indexes AST, TBiL, and ALT between the two groups (*p* = 0.331, *p* = 0.198, *p* = 0.479). After treatment, AsT, TBiL, and ALT levels were significantly increased in both groups (*p* < 0.001). However, the values of these liver function indicators were still within the normal range, as shown in Table 4.

**Table 4.** **Comparison of liver function indexes between the two groups ($\bar{x}$ ± s)**.

Group	AST (U/L)		TBiL (µmoL/L)		ALT (U/L)	
	Before Treatment	After Treatment	Before Treatment	After Treatment	Before Treatment	After Treatment
Observation group (n = 36)	27.83 ± 4.61	37.69 ± 4.80^*^	10.04 ± 1.82	16.35 ± 1.96^*^	26.16 ± 4.03	35.73 ± 3.66^*^
Control group (n = 36)	26.73 ± 4.92	37.06 ± 4.26^*^	10.68 ± 2.33	16.38 ± 1.95^*^	25.44 ± 4.54	35.13 ± 4.89^*^
*p*-value	0.331	0.558	0.198	0.948	0.479	0.558

Compared with before treatment, ^*^p < 0.001. AST, aspartate aminotransferase; TBiL, total bilirubin; ALT, alanine aminotransferase.

### 3.6 Comparison of Inflammatory Cytokine Levels between the Two Groups

As shown in Table 5, serum levels of TNF-α, IL-6, and IL-2 in both groups after treatment were lower than before treatment (*p* < 0.001). After treatment, the levels of TNF-α, IL-6, and IL-2 in the observation group were significantly lower than those in the control group (*p* < 0.001).

**Table 5.** **Comparison of inflammatory cytokine levels between the two groups ($\bar{x}$ ± s)**.

Group	TNF-α (µg/L)		IL-6 (µg/L)		IL-2 (µg/L)	
	Before Treatment	After Treatment	Before Treatment	After Treatment	Before Treatment	After Treatment
Observation group (n = 36)	6.03 ± 1.34	3.96 ± 0.24^*#^	43.78 ± 5.38	31.76 ± 3.95^*#^	38.37 ± 3.54	25.96 ± 2.95^*#^
Control group (n = 36)	6.45 ± 1.36	4.73 ± 0.21^*^	44.24 ± 4.86	38.63 ± 4.42^*^	38.82 ± 3.41	30.93 ± 3.26^*^
*p*-value	0.191	<0.001	0.705	<0.001	0.585	<0.001

Compared with before treatment, ^*^p < 0.001; compared with the control group, ^#^*p* < 0.001. TNF-α, tumor necrosis factor-α; IL-6, interleukin-6; IL-2, interleukin-2.

### 3.7 Comparison of HAMA and HAMD Scores between the Two Groups

Before treatment, there were no significant differences in HAMA and HAMD scores between the two groups (*p* = 0.856, *p* = 0.344). After treatment, HAMA and HAMD scores in both groups were significantly decreased (*p* < 0.001), and in the observation group they were significantly lower than in the control group (*p* < 0.001), as shown in Table 6.

**Table 6.** **Comparison of HAMA and HAMD scores between the two groups ($\bar{x}$ ± s)**.

Group	HAMA (score)		HAMD (score)	
	Before Treatment	After Treatment	Before Treatment	After Treatment
Observation group (n = 36)	18.42 ± 2.22	8.89 ± 1.42^*#^	19.21 ± 1.48	7.81 ± 1.45^*#^
Control group (n = 36)	18.33 ± 1.97	13.74 ± 1.87^*^	19.54 ± 1.46	13.48 ± 1.83^*^
*p*-value	0.856	<0.001	0.344	<0.001

Compared with before treatment, ^*^p < 0.001; compared with the control group, ^#^*p* < 0.001. HAMA, Hamilton Anxiety Scale; HAMD, Hamilton Depression Scale.

### 3.8 Comparison of Differences in Scores and Percentage Changes between Groups

Differences in scores and percentage changes in measurements were compared between the two groups before and after treatment. Differences in scores for material life, physical function, psychological function, social function, and HAMA and HAMD were found. The differences in scores in the observation group were greater than those in the control group (*p* <0.001). The percentage changes of neural function related factors and inflammatory cytokines were greater in the observation group than in the control group (*p* < 0.001). There was no statistical significance in the percentage changes of AST, TBiL, and ALT levels between the two groups (*p* = 0.673, *p* = 0.574, *p* = 0.787). Table 7 shows this.

**Table 7.** **Comparison of differences in scores and percentage changes between groups ($\bar{x}$ ± s)**.

	Observation group (n = 36)	Control group (n = 36)	*p*-value
Material life (score)	8.71 ± 1.05	3.97 ± 0.56	<0.001
Physical function (score)	8.64 ± 0.96	4.05 ± 0.59	<0.001
Psychological function (score)	9.07 ± 1.18	4.70 ± 0.62	<0.001
Social function (score)	11.13 ± 1.47	5.89 ± 0.73	<0.001
NSE (%)	49.83 ± 9.37	31.67 ± 7.84	<0.001
NRG1 (%)	71.35 ± 14.72	42.79 ± 9.06	<0.001
AST (%)	33.97 ± 7.41	33.24 ± 7.20	0.673
TBiL (%)	61.84 ± 12.88	60.15 ± 12.49	0.574
ALT (%)	34.55 ± 7.92	34.06 ± 7.42	0.787
TNF-α (%)	45.77 ± 8.43	26.17 ± 6.75	<0.001
IL-6 (%)	29.48 ± 6.79	13.68 ± 3.31	<0.001
IL-2 (%)	31.76 ± 6.85	18.59 ± 3.92	<0.001
HAMA (score)	10.07 ± 1.67	4.52 ± 0.73	<0.001
HAMD (score)	11.85 ± 1.79	5.84 ± 0.82	<0.001

### 3.9 Adverse Reactions

During the treatment, one case of dizziness occurred in the observation group, and one case of headache and two cases of dizziness occurred in the control group. After the treatment, all patients were cured and there were no other complications or obvious side effects.

## 4. Discussion

SCZ is a mental disease that causes relatively serious cognitive dysfunction that affects the normal functions of patients in terms of will, thinking, cognition, and social functioning, with a high recurrence rate. The quality of life in patients with SCZ is lower than that of the general population or those with other chronic diseases [16]. Antipsychotic drugs are often used in clinical treatment, and remarkable results have been achieved [17].

Paliperidone, the primary metabolite of risperidone, has an extra hydroxyl group in its structure and less drug interaction, which can significantly improve the social function of patients with good safety [18]. On the basis of paliperidone therapy, seeking other effective drug combination therapy is of great significance to enhance the therapeutic effect in SCZ patients. Sertraline can act as a dopamine blocker, which can improve the attention, energy, anxiety, depression, and other symptoms of SCZ patients and bring significant clinical benefits, thereby alleviating their cognitive symptoms. Sertraline is a safe and well-tolerated antipsychotic drug [19]. Compared to other psychotropic medications, sertraline exhibits lower activating effects, and among other 5-hydroxytryptamine reuptake inhibitor, it has minimal metabolic interactions with other drugs [20]. NSE is a glycolytic pathway enzyme, which is normally present at low levels. When neurons are injured, they overflow into the intercellular space and cerebrospinal fluid, thus entering the peripheral blood circulation of patients, resulting in an increase in NSE level [21]. NRG1 is a protein containing epidermal growth factor-like domain, which can promote normal brain development through regenerative pathways and accelerate nerve cell proliferation [22]. Research suggests that NRG1 has a potential role in the neural developmental mechanisms of SCZ [23]. Chesworth R *et al*. [24] found that NRG1 has a protective role in the pathogenesis of SCZ. Our research has shown that paliperidone combined with sertraline can effectively relieve the level of serum neuregulin, and the treatment effect is better in patients with SCZ. Wesołowska A *et al*. [25] found that paliperidone has the effect of promoting cognitive, antioxidant, and anti-inflammatory activity. Sertraline combined therapy can also effectively reduce the occurrence of adverse effects [26].

AsT, TBiL, and ALT are important indicators for detecting liver function. Once liver metabolic function is impaired, TBiL level increases, while liver cell injury and necrosis, and ALT and AST levels increase. Studies have shown that paliperidone combined with sertraline does not cause liver or kidney function damage in patients with SCZ and has only a slight impact on patients. Paliperidone has minimal liver metabolism compared with other antipsychotics and can be used safely in cases of liver injury [27]. Serum inflammatory factors are significantly correlated with SCZ. In this study, the levels of TNF-α, IL-6, and IL-2 in the observation group after treatment were significantly lower than those in the control group, indicating that paliperidone combined with sertraline can inhibit the inflammatory response and improve clinical symptoms in patients with SCZ. In addition, SCZ patients mostly have symptoms such as compulsion, mania, anxiety, and depression, and sertraline is a commonly used antidepressant drug [28] that has a good effect on SCZ patients with positive and negative symptoms, with few adverse reactions. Paliperidone combined with sertraline can improve the negative emotional effects of SCZ patients, so as to ensure the quality of life of patients, and the curative effect is positive.

## 5. Conclusions

In summary, paliperidone combined with sertraline can effectively alleviate the serum neuregulin level in patients with SCZ, improve the negative emotional effects of patients, and does not cause liver or kidney function damage. The clinical efficacy and safety of this combination are promising, as it can help patients to recover their social function quickly and is suitable for clinical application. The shortcoming of this study lies in the small sample size, which needs to be expanded to further confirm the research results.

## Availability of Data and Materials

The datasets used and/or analyzed during the current study are available from the corresponding author on reasonable request.

## References

[1] Bansal S, Bae GY, Frankovich K, Robinson BM, Leonard CJ, Gold JM (2020). Increased repulsion of working memory representations in schizophrenia. *Journal of Abnormal Psychology*.

[2] McCutcheon RA, Reis Marques T, Howes OD (2020). Schizophrenia-An Overview. *JAMA Psychiatry*.

[3] Richetto J, Meyer U (2021). Epigenetic Modifications in Schizophrenia and Related Disorders: Molecular Scars of Environmental Exposures and Source of Phenotypic Variability. *Biological Psychiatry*.

[4] Weinhold SL, Lechinger J, Ittel J, Ritzenhoff R, Drews HJ, Junghanns K (2022). Dysfunctional Overnight Memory Consolidation in Patients with Schizophrenia in Comparison to Healthy Controls: Disturbed Slow-Wave Sleep as Contributing Factor?. *Neuropsychobiology*.

[5] Valsecchi P, Barlati S, Garozzo A, Deste G, Nibbio G, Turrina C (2019). Paliperidone palmitate in short- and long-term treatment of schizophrenia. *Rivista Di Psichiatria*.

[6] McCutcheon RA, Krystal JH, Howes OD (2020). Dopamine and glutamate in schizophrenia: biology, symptoms and treatment. *World Psychiatry*.

[7] Buck SA, Quincy Erickson-Oberg M, Logan RW, Freyberg Z (2022). Relevance of interactions between dopamine and glutamate neurotransmission in schizophrenia. *Molecular Psychiatry*.

[8] Na H, Yan LH, Liu FH (2019). Effects of Mengshi Guntan Wan combined with perperone on serum biochemical indexes in patients with schizophrenia. *Drug Evaluation Research*.

[9] Girdler SJ, Confino JE, Woesner ME (2019). Exercise as a Treatment for Schizophrenia: A Review. *Psychopharmacology Bulletin*.

[10] Pu ZP, Li GR, Zou ZP, Tao F, Hu SH (2019). A Randomized, 8-Week Study of the Effects of Extended-Release Paliperidone and Olanzapine on Heart Rate Variability in Patients with Schizophrenia. *Journal of Clinical Psychopharmacology*.

[11] Liu PP, Liu XJ, Huang ZC, Liu R, Liu Y (2022). Clinical study of Tianmeng Oral Liquid combined with sertraline on improving cognitive function, anxiety and sleep disorders in patients with cerebral hypofunction. *Chinese Traditional and Herbal Drugs*.

[12] Lu R, Lu M (1999). Analysis of symptoms of acute and chronic schizophrenia according to ICD-10. *Journal of Clinical Psychiatry*.

[13] Xiong J (2013). Diagnosis and treatment of schizophrenia. *Chinese Modern Drug Application*.

[14] Seow LSE, Lau JH, Abdin E, Verma SK, Tan KB, Subramaniam M (2023). Mapping the schizophrenia quality of life scale to EQ-5D, HUI3 and SF-6D utility scores in patients with schizophrenia. *Expert Review of Pharmacoeconomics & Outcomes Research*.

[15] Chang X, Zhao Q, Zhao X, Yang X, Chang Y (2020). Clinical efficacy of aripiprazole combined with Sertraline in the treatment of schizophrenia with depression. *Shaanxi Medical Journal*.

[16] Lin C, Wan X, Zhang R, Yang X, Liu Y (2023). Quality of life and its influencing factors in patients with schizophrenia. *Zhong Nan Da Xue Xue Bao. Yi Xue Ban = Journal of Central South University. Medical Sciences*.

[17] Ishii J, Kodaka F, Miyata H, Yamadera W, Seto H, Higuchi H (2022). Association between functional recovery and medication adherence in schizophrenia. *Neuropsychopharmacology Reports*.

[18] Minwalla HD, Wrzesinski P, Desforges A, Caskey J, Wagner B, Ingraffia P (2021). Paliperidone to Treat Psychotic Disorders. *Neurology International*.

[19] DeVane CL, Liston HL, Markowitz JS (2002). Clinical pharmacokinetics of sertraline. *Clinical Pharmacokinetics*.

[20] Lang X, Xue M, Zang X, Wu F, Xiu M, Zhang X (2023). Efficacy of low-dose risperidone in combination with sertraline in first-episode drug-naïve patients with schizophrenia: a randomized controlled open-label study. *Journal of Translational Medicine*.

[21] Shang Y, Qi F, Zheng Z, Yang G, Fei F, Guo Q (2022). Effect of bilateral paravertebral nerve block on cognitive function in elderly patients undergoing radical gastrectomy for gastric cancer: a prospective randomized double-blind controlled trial. *BMC Aesthesiology*.

[22] Zieba J, Morris MJ, Weickert CS, Karl T (2020). Behavioural effects of high fat diet in adult Nrg1 type III transgenic mice. *Behavioural Brain Research*.

[23] Rajasekaran A, Shivakumar V, Kalmady SV, Parlikar R, Chhabra H, Prabhu A (2020). Impact of NRG1 HapICE gene variants on digit ratio and dermatoglyphic measures in schizophrenia. *Asian Journal of Psychiatry*.

[24] Chesworth R, Rosa-Porto R, Yao S, Karl T (2021). Sex-specific sensitivity to methamphetamine-induced schizophrenia-relevant behaviours in neuregulin 1 type III overexpressing mice. *Journal of Psychopharmacology (Oxford, England)*.

[25] Wesołowska A, Jastrzębska-Więsek M, Cios A, Partyka A (2020). The preclinical discovery and development of paliperidone for the treatment of schizophrenia. *Expert Opinion on Drug Discovery*.

[26] Zhu C, Guan X, Wang Y, Liu J, Kosten TR, Xiu M (2022). Low-Dose Ziprasidone in Combination with Sertraline for First-Episode Drug-Naïve Patients with Schizophrenia: a Randomized Controlled Trial. *Neurotherapeutics: the Journal of the American Society for Experimental NeuroTherapeutics*.

[27] Vats A, Nair AR, Bandhu AK, Koirala D, Pallapothu MR, Quintana Mariñez MG (2023). Treatment of Patients with Schizophrenia and Comorbid Chronic Hepatitis With Paliperidone: A Systematic Review. *Cureus*.

[28] Ch S, Sudha S, Reddy CG, T P, Ksbs KS, Dasari P (2022). A Comparative Study on Safety and Efficacy of Desvenlafaxine Versus Sertraline in Depression. *Cureus*.

